# Central Nervous Insulin Administration before Nocturnal Sleep Decreases Breakfast Intake in Healthy Young and Elderly Subjects

**DOI:** 10.3389/fnins.2017.00054

**Published:** 2017-02-08

**Authors:** João C. P. Santiago, Manfred Hallschmid

**Affiliations:** ^1^Institute of Medical Psychology and Behavioral Neurobiology, University of TübingenTübingen, Germany; ^2^German Center for Diabetes ResearchTübingen, Germany; ^3^Institute for Diabetes Research and Metabolic Diseases of the Helmholtz Center Munich at the University of TübingenTübingen, Germany

**Keywords:** intranasal insulin, food intake behavior, sleep, aging, sex distribution

## Abstract

Peripheral insulin acts on the brain to regulate metabolic functions, in particular decreasing food intake and body weight. This concept has been supported by studies in humans relying on the intranasal route of administration, a method that permits the direct permeation of insulin into the CNS without substantial absorption into the blood stream. We investigated if intranasal insulin administration before nocturnal sleep, a period of reduced metabolic activity and largely absent external stimulation, affects food intake and energy turnover on the subsequent morning. Healthy participants who were either young (16 men and 16 women; mean age ± SEM, 23.68 ± 0.40 years, mean BMI ± SEM, 22.83 ± 0.33 kg/m^2^) or elderly (10 men, 9 women; 70.79 ± 0.81 years, 25.27 ± 0.60 kg/m^2^) were intranasally administered intranasal insulin (160 IU) or placebo before a night of regular sleep that was polysomnographically recorded. Blood was repeatedly sampled for the determination of circulating glucose, insulin, leptin and total ghrelin. In the morning, energy expenditure was assessed via indirect calorimetry and subjects were offered a large standardized breakfast buffet from which they could eat *ad libitum*. Insulin compared to placebo reduced breakfast size by around 110 kcal (1,054.43 ± 50.91 vs. 1,162.36 ± 64.69 kcal, *p* = 0.0095), in particular decreasing carbohydrate intake (502.70 ± 25.97 vs. 589.82 ± 35.03 kcal, *p* = 0.0080). This effect was not dependent on sex or age (all *p* > 0.11). Sleep architecture, blood glucose and hormonal parameters as well as energy expenditure were not or only marginally affected. Results show that intranasal insulin administered to healthy young and elderly humans before sleep exerts a delayed inhibitory effect on energy intake that is not compensated for by changes in energy expenditure. While the exact underlying mechanisms cannot be derived from our data, findings indicate a long-lasting catabolic effect of central nervous insulin delivery that extends across sleep and might be of particular relevance for potential therapeutic applications.

## Introduction

Eating behavior is tightly regulated by central nervous circuitries that receive hormonal feedback on body fat stores and nutritional status from the body periphery (Morton et al., 2014). In addition to the adipocyte-derived hormone leptin, insulin is one of the major peripheral signals that contribute to the central nervous control of ingestive behavior. Both leptin and insulin circulate in direct proportion to the size of body fat stores and reach the CNS via active, saturable transport mechanisms across the blood-brain barrier (Baura et al., 1993; Schwartz et al., 1996; William and Banks, 2001). Studies in animals (Woods et al., 1979; McGowan et al., 1992; Air et al., 2002) and humans (Benedict et al., 2008; Hallschmid et al., 2012) have conclusively shown that insulin administered directly to the brain reduces food intake, independent of its peripheral glucoregulatory action. In humans, the inhibitory effect of central nervous insulin on food intake has been mainly investigated by means of the intranasal route of peptide administration (Hallschmid et al., 2004a, 2012; Benedict et al., 2008), a non-invasive method of substance delivery to the brain that largely bypasses the blood-brain barrier (Born et al., 2002; Dhuria et al., 2010). Intranasal administration of 160 IU insulin to healthy, fasted young subjects reduced calorie intake in male, but not female participants (Benedict et al., 2008). Accordingly, intranasal insulin treatment (4 × 40 IU/day) for 8 weeks resulted in loss of body weight and body fat in men but not in women (Hallschmid et al., 2004a). Still, young women who intranasally received 160 IU insulin after a standardized lunch displayed an enhancement of post-prandial satiety and a reduction in the intake of palatable snacks (Hallschmid et al., 2012). Ample evidence for a distinct effect of insulin on food intake-regulatory networks has also been found in related neuroimaging studies (see Heni et al., 2015; Kullmann et al., 2016 for reviews).

Sleep has turned out to be an important factor in the maintenance of energy homeostasis and the regulation of food intake (St-Onge et al., 2016). Habitually short sleep duration is associated with increased body weight (Magee and Hale, 2012; Vgontzas et al., 2014) and a more pronounced risk of impairments in glucose homeostasis (Gangwisch et al., 2007; Cappuccio et al., 2010). Fittingly, individuals exposed to acute sleep deprivation tend to consume more food on the subsequent day (Brondel et al., 2010), to reduce their physical activity (Schmid et al., 2009) and to display a deterioration in glucoregulation (Schmid et al., 2011). It has been proposed that increased energy expenditure due to sleep loss is overcompensated by an exaggerated increase in energy intake, resulting, on the long run, in a higher risk of obesity and related metabolic impairments (Penev, 2012; Schmid et al., 2015). We have previously shown that insulin applied to the CNS before nocturnal sleep increases growth hormone concentrations during early sleep and impacts memory function on the subsequent day (Feld et al., 2016), indicating that central nervous insulin signaling is relevant for sleep-associated neuroendocrine regulation. In the present study, we investigated the effect of intranasal insulin administered before sleep on eating behavior on the subsequent morning. We assumed that the acute enhancement of brain insulin signaling during sleep, i.e., a period of reduced metabolic activity and largely absent external input, exerts a delayed but discernible attenuating effect on breakfast intake, i.e., calorie consumption immediately following the sleep period. This hypothesis was tested in a group of healthy young men and women, thereby enabling the detection of potential sex differences. Considering reports that food-cue elicited changes in brain activity in response to a meal decrease with advancing age (Cheah et al., 2013), we moreover included a group of healthy elderly participants in order to investigate if age is a relevant modulatory factor in this context.

## Methods and materials

### Participants

Thirty-two healthy young subjects (16 men and 16 women, mean age ± SEM, 23.68 ± 0.40 years) and 19 elderly participants (10 men and 9 women, 70.79 ± 0.81 years) were recruited from the community for this study. All young subjects were normal-weight (BMI, 22.83 ± 0.33 kg/m^2^, *p* = 0.49 for men vs. women) while the elderly participants were normal- or mildly overweight (25.27 ± 0.60 kg/m^2^, *p* = 0.70 for men vs. women; *p* < 0.001 for young vs. elderly participants). All subjects were non-smokers. The women in the young group were taking oral contraceptives (estrogen dominant, single-phase; Valette, Jenapharm, Jena, Germany), but were otherwise free of medication, as were the men. Clinical examination excluded previous illness prior to inclusion in the study. In order to restrict the burden of experimental participation for the elderly subjects, some assessments (in particular energy expenditure and continuous heart rate monitoring) were omitted and less blood parameters were determined in this group. Written informed consent was obtained from all subjects and the study conformed to the Declaration of Helsinki and was approved by the local ethics committee.

### Study design and procedure

The experiments were conducted according to a placebo-controlled, double-blind, within-subject crossover design. All participants took part in two experimental sessions which were identical except for the intranasal administration of insulin (Actrapid®, Novo Nordisk, Bagsværd, Denmark) or placebo (vehicle). Sessions were performed in a balanced order, i.e., half of the sample received first placebo and then the active agent, with the reversed order for the other half of the sample. In addition, participants spent an adaption night in the sleep lab (i.e., including the placement of electrodes for polysomnographic recordings), with at least a 24-h delay between adaptation and the first experiment. Experimental sessions were scheduled to be apart as close to 28 days as possible, ensuring that the young women were tested during the same phase of contraceptive intake in both sessions.

Subjects were instructed not to take naps and not to engage in intense physical activities on experimental days. They were told to abstain from caffeine and to follow their usual dinner routines around 1800–1900 h. Participants arrived at the sleep lab at 2000 h. Adherence to the instructions for the experimental day was confirmed and an intravenous catheter was placed in a vein of the dominant arm. At 2120 h, participants underwent a memory test battery (see Feld et al., 2016, for details and respective results in the group of young subjects) before receiving intranasal insulin or placebo via sixteen 0.1-ml puffs (8 per nostril) in 1-min intervals, amounting to a total dose of 1.6 ml insulin (160 IU) or placebo at 2220 h. This dose was chosen in order to enable comparisons with previous studies on the role of central nervous insulin in the acute regulation of food intake (Benedict et al., 2008; Hallschmid et al., 2012). Subjects went to bed at 2300 h for 8 h of polysomnographically recorded sleep, resulting in an overnight fast of at least 12 h in all subjects. Subjects were awakened at approximately 0700 h; care was taken not to wake participants up from rapid eye movement (REM) sleep or slow wave sleep. In the young subjects, heart rate was recorded throughout the night and energy expenditure was assessed at 0710 h in the morning. At 0815 h, breakfast was offered to all subjects. Throughout the session, blood was repeatedly sampled, without disturbing the participants, from an adjacent room with a thin plastic tube attached to the catheter for the determination of relevant parameters. Venous patency was maintained with a NaCl 0.9% drip.

### Assessment of breakfast intake

Participants were offered a standardized test buffet of approximately 4,550 kcal at 0815 h and were allowed to eat ad-libitum and undisturbed for 30 min (see Table 1 for a list of ingredients). All ingredients were weighed before and after eating to calculate the net amount consumed. Participants were allowed to take any leftovers with them in order to prevent them from overconsumption. They were told that this breakfast was scheduled to fill the gap between cognitive tests and provide them with the possibility to follow their usual breakfast routine, so that the experimental nature of this buffet remained undisclosed. One male and three female participants of the young group and one male and two female participants of the elderly group abstained from eating breakfast and were excluded from food intake analyses. All participants rated their hunger, thirst and tiredness on visual analog scales (VAS) before breakfast.

**Table 1:** **Composition of the test breakfast buffet**.

**Food**	**Weight (g)**	**Energy (kcal)**	**Carbohydrate (g)**	**Fat (g)**	**Protein (g)**
**NEUTRAL**					
Whole milk	750	491	36	26.3	24.8
Buns	240	275	122.4	3.4	6.3
Whole wheat bread	165	360	71	2.3	12
White bread	30	72	14.6	0.4	2.2
Butter	120	928	0.7	99.8	0.8
**SWEET**					
Orange juice	400	173	36	1	4
Strawberry milk	200	167	18.2	6.8	7.4
Apple	195	104	22.2	1.2	0.6
Orange	180	72	15	0.4	1.8
Banana	179	168	38.3	0.4	2
Pear	140	78	17.4	0.4	0.7
Fruit curd	125	140	19.3	3.3	7.7
Vanilla pudding	125	134	20.8	3.8	3.5
Tangerine	80	35	8.2	0	0.5
Strawberry jam	50	147	35.8	0.1	0.1
Hazelnut spread	40	218	21.6	12.8	2.8
Honey	40	123	30	0	0.1
Sugar	24	98	24	0	0
**HEARTY**					
Sliced cheese	100	374	0.1	29.2	25.5
Poultry sausage	40	74	0.1	4.3	8.3
Cream cheese (herbs)	40	124	1	11.6	3.2
Cervelat sausage	34	120	0.1	10.2	6.1
Cream cheese	33	87	0.6	7.8	3
Total	3,330	4,562	553.4	225.5	123.4

*All values are rounded to the closest decimal*.

### Energy expenditure

In the participants of the young group, energy expenditure was measured at 0710 h via indirect calorimetry using a ventilated-hood system (Deltatrac II, MBM-200 Metabolic Monitor; Datex-Engström Deutschland, Achim, Germany). Before each use, the device was calibrated with Quick Cale calibration gas to 5% CO_2_ and 95% O_2_. Due to technical failures, assessments were not possible in two subjects.

### Blood parameters

Blood samples for the determination of blood glucose levels and circulating concentrations of hormones were obtained before intranasal insulin administration and repeatedly throughout the night. Blood glucose was determined immediately after each blood draw (HemoCue Glucose 201 Analyzer, HemoCue AB, Ångelholm, Schweden). The remaining samples were centrifuged and serum and plasma were frozen at −80°C for later analyses. Insulin concentrations were determined in young and elderly participants (Insulin ELISA Kit, Dako, Glostrup, Denmark). In the young participants, plasma concentrations of total ghrelin (RIA; Linco Research, St. Charles, MO; sensitivity 93 pg/ml, intra-assay and inter-assay CV, 10 and 17.8%) and serum concentrations of leptin (RIA; Linco Research, St. Charles, MO; sensitivity, 0.5 ng/ml, intra-assay and inter-assay CV, 8.3% and 6.2%) were measured at time-points of relevance throughout the night.

### Polysomnography and heart rate

EEG was recorded continuously from electrodes (Ag-AgCl) placed at C3 and C4 according to the 10-20 System and referenced to two coupled electrodes attached at the mastoids. EEG signals were filtered between 0.16 and 35 Hz and sampled at a rate of 200 Hz using an EEG amplifier system (BrainAmp DC, BrainProducts GmbH, Munich, Germany). Additionally, eye movements and muscle tone were recorded by electrodes placed diagonally above the left and below the right eye and electrodes attached to the chin, respectively. Sleep EEG scoring was carried out independently by two experienced technicians who were blind to the assigned treatment. Sleep architecture was determined according to standard polysomnographic criteria using EEG recordings from C3 and C4, diagonal EOG and chin EMG (Rechtschaffen and Kales, 1968). For each night, total sleep time, i.e., the time between the first detection of transition from sleep stages 1 to 2 and lights on, was used to calculate relative time spent in the different sleep stages, i.e., wake, REM sleep and NonREM sleep stages 1–4. Heart rate was recorded by electrocardiography (Actiheart, CamNtech, Boerne TX USA) in the young subjects during sleep until shortly after awakening. In the elderly subjects, it was monitored before and after sleep.

### Statistical analyses

R 3.3.1 (R Core Team, 2016) was used for statistical analyses. We used *lme4* (Bates et al., 2015) to build linear mixed-effects models to compensate for missing blood values in some cases (less than four per experiment). Main effects were tested for significance using likelihood-ratio tests with Satterthwaite approximations to degrees of freedom. For the indirect calorimetry data, we used condition (placebo or insulin) and sex as fixed effects and random intercepts for subjects. For breakfast consumption, we built an initial model using condition, sex, age group (young, elderly), macronutrient (carbohydrate, fat, protein) and interactions between condition, sex and macronutrient, condition and sex, condition and age and sex and macronutrient as fixed effects, random slopes for macronutrient and random intercepts for subjects. The inclusion of a random slope takes into account individual food preferences of each subject, thus optimally capturing between-subject differences. To evaluate intake by food type (hearty, neutral, sweet), we replaced macronutrient with food type in the previous model. For the VAS we built a model with fixed effects for treatment, sex, age group and random intercepts for subjects. We used *lsmeans* (Lenth, 2016) to run *post-hoc* comparisons with multivariate *t* adjustment. Levene's test for homogeneity of variance was used to test for equality of variances in our factors of interest, with only sex showing a deviation (*p* < 0.01; *p* ≥ 0.12 for age and treatment). For analyses of blood parameters, the fixed factors sex, condition and time were used and areas under the curve (AUC) according to the trapezoidal rule were calculated. Results are presented as means ± SEM. A *p* < 0.05 was considered significant.

## Results

### Breakfast intake and hunger ratings

**Figure 1:**
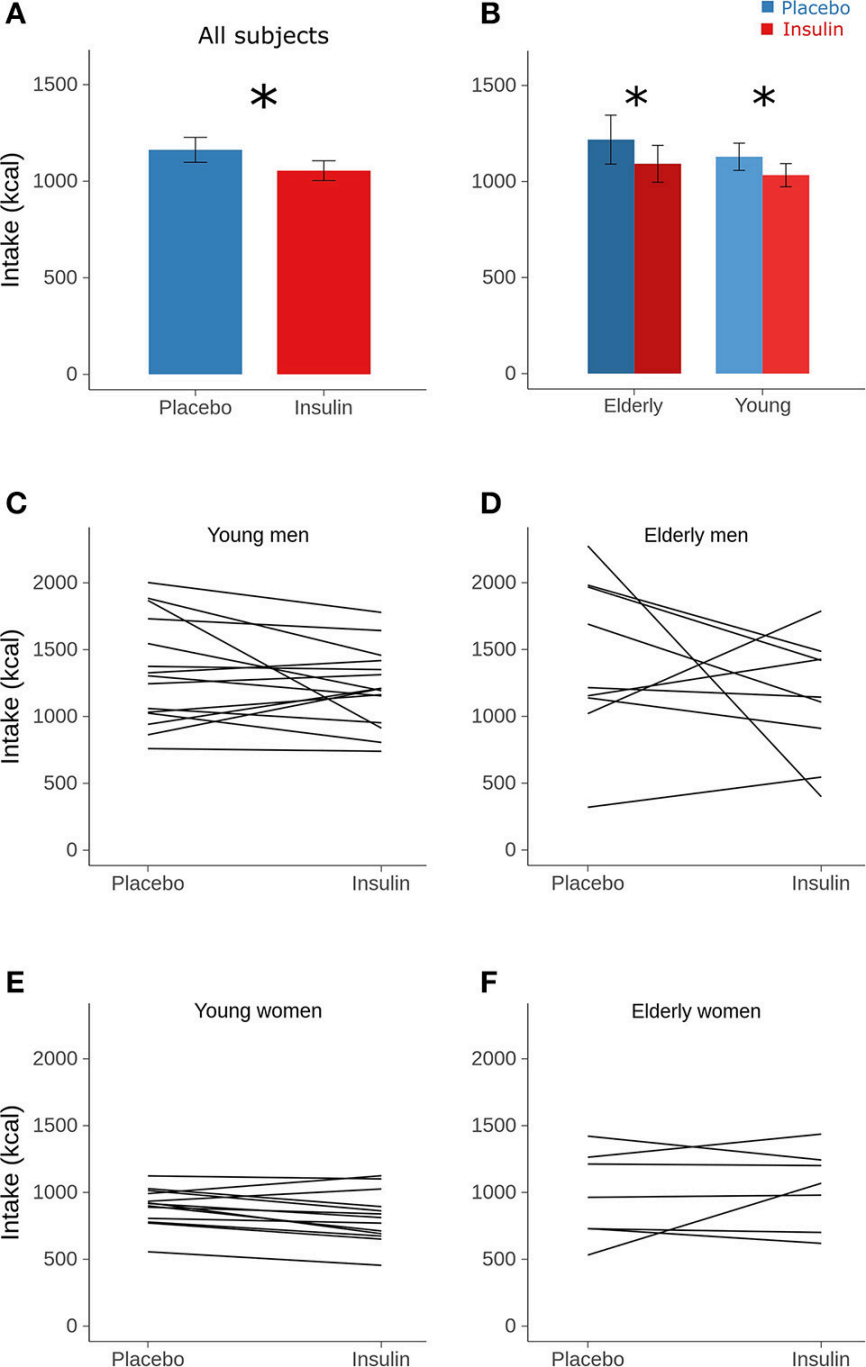
**Calorie intake**. Mean (± SEM) calorie intake from the breakfast buffet assessed in the morning after intranasal administration of placebo (vehicle; blue bars) and insulin (160 IU; red bars) at 2220 h of the preceding evening in **(A)** all subjects and **(B)** according to age groups. **(C–F)** Individual calorie intake from the breakfast buffet in the placebo (left) and the insulin condition (right) in respectively, the young and elderly men and women. Individual values of both sessions are connected by lines. Note that omitting the male subjects showing the largest insulin effect yielded *p*-values for the factor condition of 0.015 and 0.079 in the groups of young and, respectively, elderly subjects. *n* = 14 young and 9 elderly men, and 14 young and 7 elderly women; ^*^p < 0.05 for comparisons between conditions (least-square means with multivariate *t* adjustment).

Intranasal insulin administration before sleep reduced breakfast intake by 110 kcal or around 9% (Table 2 and Figure 1A and Supplementary Figure 1). This hypophagic effect of insulin primarily concerned carbohydrate intake, whereas consumption of fat and protein was not affected [*F*_(2, 170)_ = 3.23, *p* = 0.042 for treatment × macronutrient; Table 2]. The insulin effect was modified neither by sex nor age (all *p* > 0.11 for interaction with treatment; Figure 1B) and did not specifically affect individual food types [hearty, neutral, sweet; *F*_(2, 233)_ = 0.71, *p* = 0.49 for treatment × food type]. Independent of insulin treatment, men consumed more energy than women [1,276.23 ± 86.76 vs. 913.23 ± 50.64 kcal, *F*_(1, 43)_ = 17.34, *p* = 0.0001; Figures 1C–F], in particular fat [501.01 ± 43.50 vs. 330.25 ± 29.72 kcal, *t*_(54)_ = 4.33, *p* = 0.0008; *p* > 0.05 for carbohydrates and protein; *F*_(2, 54)_ = 4.13, *p* = 0.02 for sex × macronutrient]. Men also ate more neutral-taste foods than women [248.74 ± 43.48 vs. 168.80 ± 22.92 kcal, *t*_(146)_ = 5.73, *p* < 0.0001; *p* > 0.84 for hearty and sweet foods]. Calorie intake of young and elderly participants was generally comparable [1,080.77 ± 65.85 vs. 1,156.91 ± 111.48 kcal, *F*_(1, 44)_ = 0.64, *p* = 0.43; *F*_(1, 55)_ = 0.69, *p* = 0.41 for sex × age; Figures 1C–F] and also did not differ regarding food types [*F*_(2, 231)_ = 0.53, *p* = 0.59]. Age and macronutrient showed a significant interaction [*F*_(2, 54)_ = 4.38, *p* = 0.02] that, however, did not yield significant age-dependent effects on macronutrient intake (all *p* > 0.17).

**Table 2:** **Consumption from the breakfast buffet**.

	**All subjects**			**Men**			**Women**			**Young subjects**			**Elderly subjects**		
	**Insulin**	**Placebo**	***p***	**Insulin**	**Placebo**	***p***	**Insulin**	**Placebo**	***p***	**Insulin**	**Placebo**	***p***	**Insulin**	**Placebo**	***p***
Overall consumption	1,054.43 ± 50.91	1,162.36 ± 64.69	0.0095	1,188.79 ± 72.29	1,363.66 ± 97.42	0.0146	893.20 ± 54.30	932.30 ± 47.87	0.0143	1,032.90 ± 60.44	1,128.64 ± 70.78	0.0285	1,092.12 ± 96.11	1,217.89 ± 127.38	0.0210
**MACRO-NUTRIENTS**															
Carbohydrate	502.70 ± 25.97	589.82 ± 35.03	0.0079	543.82 ± 39.02	678.63 ± 55.67	0.0075	453.36 ± 30.66	488.32 ± 27.31	0.0073	531.03 ± 28.66	587.09 ± 36.26	0.001	453.12 ± 50.91	594.32 ± 72.64	0.0090
Fat	413.85 ± 28.95	430.65 ± 30.92	0.9866	485.51 ± 41.06	516.50 ± 46.48	0.9856	327.84 ± 32.46	332.54 ± 27.67	0.9853	368.75 ± 30.92	404.93 ± 33.51	0.9922	492.76 ± 55.63	473.02 ± 60.48	0.9916
Protein	137.89 ± 8.70	141.89 ± 9.15	1	159.46 ± 13.25	168.54 ± 13.29	1	112.00 ± 7.94	111.43 ± 8.68	1	133.12 ± 10.39	136.62 ± 11.28	1	146.23 ± 16.23	150.55 ± 15.74	1

*Results are means ± SEM kcal, rounded to the nearest decimal. P-values are derived from least-squares means with multivariate t adjustment*.

Overall, intranasal insulin did not alter hunger, thirst and tiredness as rated before breakfast (all *p* > 0.50), and there was no influence of sex on these values (all *p* > 0.33; Table 3). Elderly participants reported significantly lower hunger than their young counterparts [43.75 ± 3.81 vs. 67.56 ± 4.52%, *t*_(122)_ = −4.64, *p* = 0.0001], with no differences in thirst and tiredness (all *p* > 0.19; Table 3).

**Table 3:** **Visual analog scale ratings obtained before breakfast**.

	**Overall**			**Sex**			**Age group**		
	**Insulin**	**Placebo**	***p***	**Female**	**Male**	***p***	**Elderly**	**Young**	***p***
Hungry	58.96 ± 3.55	60.10 ± 3.63	1	55.84 ± 5.78	62.86 ± 4.26	0.4382	43.75 ± 3.81	67.56 ± 4.52	0.0001
Thirsty	63.00 ± 2.94	61.33 ± 3.25	0.9311	59.31 ± 4.77	64.72 ± 3.96	0.7564	54.69 ± 3.78	65.95 ± 4.17	0.1974
Tired	32.93 ± 3.17	36.98 ± 3.40	0.9837	41.14 ± 4.71	29.60 ± 4.37	0.3319	31.29 ± 4.61	36.83 ± 4.40	0.9398

*Results are means ± SEM %. p values are derived from least-square means contrasts with multivariate t adjustment*.

### Energy expenditure

**Figure 2:**
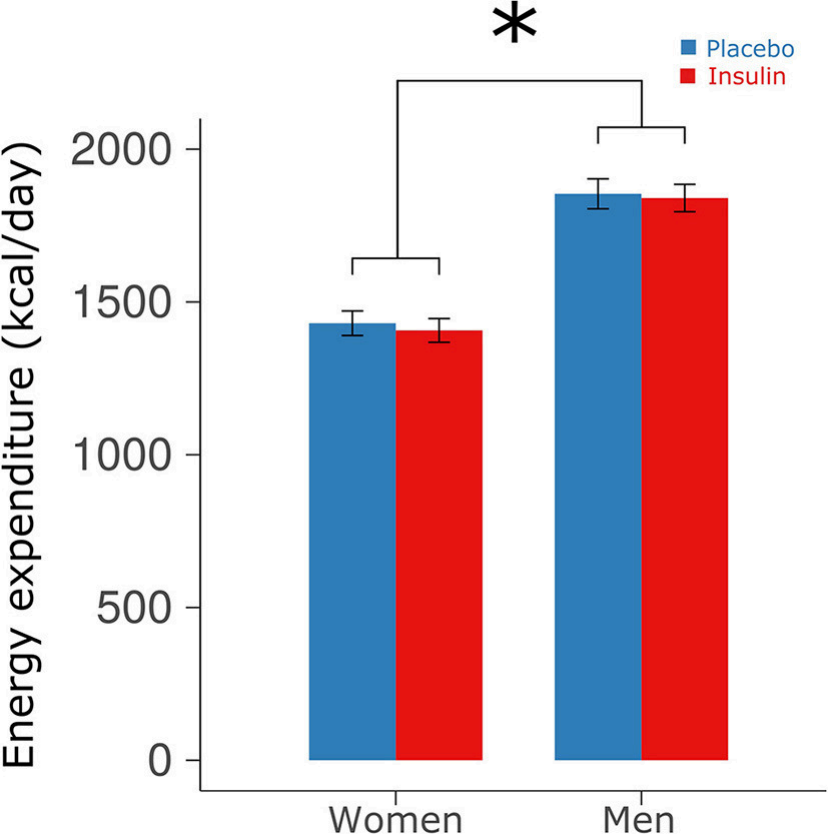
**Energy expenditure**. Mean ± SEM energy expenditure measured in the young participants via indirect calorimetry in the morning after intranasal administration of placebo (vehicle; blue bars) and insulin (160 IU; red bars) at 2220 h of the preceding evening. Calorimetry was performed in the fasted state at 0710 h before breakfast. ^*^p < 0.05 for comparisons between sexes.

Intranasal insulin administration before sleep did not affect resting energy expenditure measured in the young subjects before breakfast [1,637.96 ± 50.25 vs. 1,656.32 ± 50.87 kcal/day for insulin and placebo, respectively, *F*_(1, 29)_ = 0.971, *p* = 0.33; Figure 2] and also did not induce sex-dependent changes [*F*_(1, 28)_ = 0.07, *p* = 0.80]. Across conditions, women displayed significantly lower energy expenditure than men [1,418.63 ± 41.56 vs. 1,847.05 ± 46.24 kcal/day, *F*_(1, 28)_ = 49.27, *p* < 0.0001].

### Blood parameters

In the young participants, a transient peak in plasma insulin concentration emerged 10 min after intranasal insulin administration (111.19 ± 9.38 vs. 56.67 ± 4.79 pmol/L after placebo; *p* ≤ 0.001) that was followed by a slight dip in blood glucose values (4.38 ± 0.13 vs. 4.86 ± 0.06 mmol/L; *p* ≤ 0.01). Neither of these changes was correlated with the intranasal insulin-induced decrease in breakfast intake in the subsequent morning (*r* = −0.15, *p* = 0.54, and *r* = 0.02, *p* = 0.95, respectively, Pearson's coefficients). During the rest of the night, respective values were comparable between conditions [*F*_(6, 156)_ = 0.86, *p* = 0.65 for treatment × time; see Feld et al., 2016, for detailed results], without any statistical difference to the elderly group (*p* = 0.24 for age). Serum insulin as well as blood glucose concentrations were not affected by insulin administration in the elderly subjects (all *p* > 0.58). Across conditions, blood glucose levels were lower in elderly than young [*F*_(1, 105)_ = 14.49, *p* < 0.001 for age] and in female than male individuals [*F*_(1, 25)_ = 10.14, *p* < 0.01 for sex].

**Figure 3:**
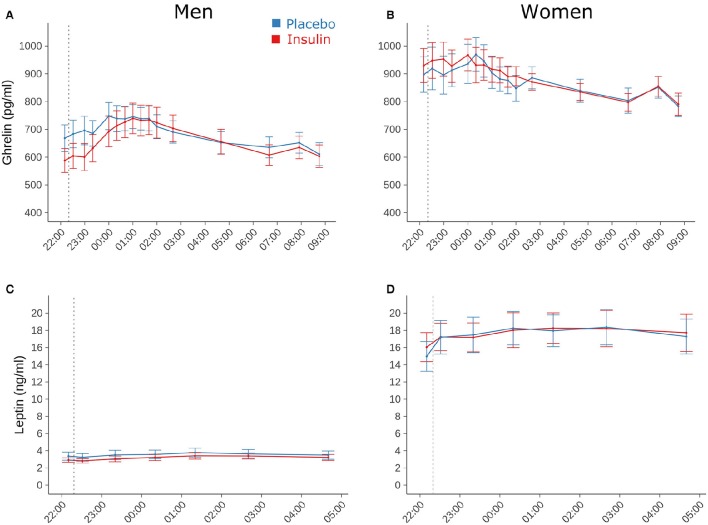
**Blood parameters**. Mean ± SEM concentrations of **(A,B)** plasma total ghrelin and **(C,D)** serum leptin measured in young male (*n* = 16; left panels) and female participants (*n* = 16; right panels) who were intranasally administered insulin (160 IU; red lines) or placebo (blue lines) at 2220 h (dotted line). Note that leptin concentrations were determined only until 0440 h in the morning.

Plasma concentrations of total ghrelin, measured only in the group of young subjects, did not differ between conditions [17,940.26 ± 830.37 vs. 17,903.28 ± 763.61 h × pg/ml for insulin and placebo, respectively; *F*_(1, 108)_ = 0.01, *p* = 0.95; *F*_(1, 909)_ = 0.0042, *p* = 0.95 for time × treatment; Figures 3A,B]. They were generally elevated in women compared to men [20,258.09 ± 915.24 vs. 15,429.69 ± 967.39 h × pg/ml, *F*_(1, 29)_ = 16.31, *p* = 0.0004]. Serum leptin concentrations in the young participants also remained unaffected by intranasal insulin [154.10 ± 23.25 vs. 156.47 ± 23.75 h × ng/ml, *F*_(1, 31)_ = 0.04, *p* = 0.85; *F*_(1, 406)_ = 0.18, *p* = 0.67 for time × treatment] and, as expected, were markedly higher in women compared to men [259.78 ± 27.57 vs. 50.69 ± 6.30 h × ng/ml, *F*_(1, 30)_ = 67.35, *p* < 0.0001, Figures 3C,D].

### Sleep and heart rate

**Figure 4:**
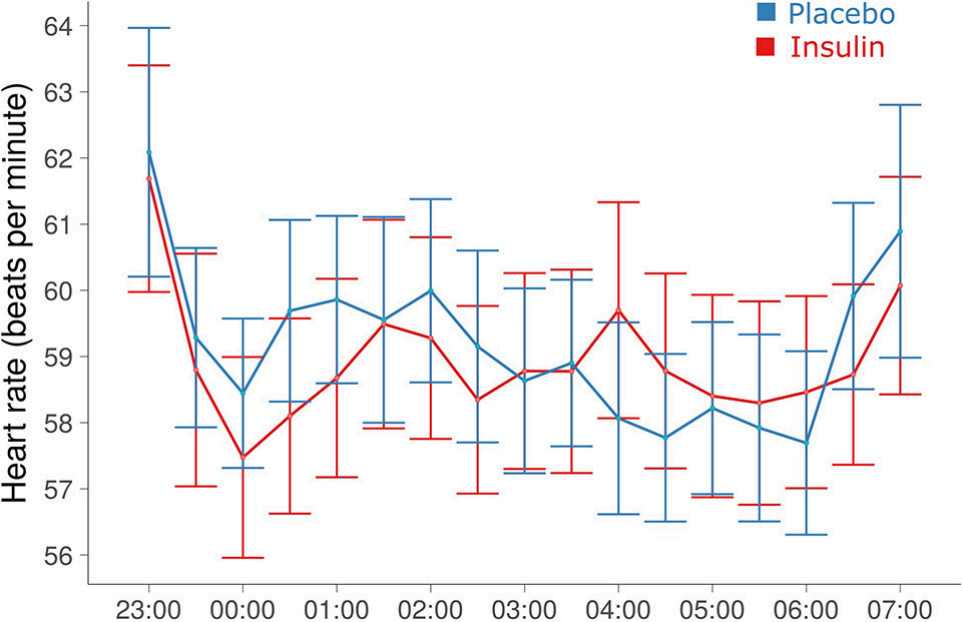
**Heart rate during the sleep period**. Mean ± SEM heart rate averaged across 30-min blocks from minute-to-minute recordings. No signs of coherent treatment effects were detected (see Results).

Total sleep time for the young participants was 461.59 ± 3.37 min in the insulin and 460.70 ± 4.57 min in the placebo condition (*p* > 0.84). Elderly participants spent comparable amounts of time asleep (456.57 ± 11.12 vs. 458.39 ± 5.68 min, *p* > 0.89; *p* > 0.57 for group effect). Intranasal insulin compared to placebo did not alter sleep latency, whole-night sleep architecture and total sleep time (all *p* > 0.29). Heart rate measured in the young subjects throughout the night was unchanged by insulin [58.77 ± 1.59 vs. 58.98 ± 1.48 bpm, *F*_(1, 29, 006)_ = 0.08, *p* = 0.77; Figure 4]. Independent of treatment, it showed a trend toward increased values in women compared to men [61.19 ± 1.96 vs. 56.63 ± 2.10 bpm; *F*_(1, 30)_ = 3.72, *p* = 0.063]. Heart rate measured in the elderly subjects before and after sleep was not modulated by intranasal insulin (all *p* ≥ 0.18).

## Discussion

We investigated whether a single dose of intranasal insulin administered to healthy young and elderly subjects before nocturnal sleep attenuates calorie intake from a large, standardized ad-libitum breakfast buffet on the subsequent day. We found that pre-sleep insulin treatment reduced breakfast consumption by around 110 kcal, which is roughly equivalent to a large banana or half a chocolate-caramel bar. This effect emerged against the background of unaltered energy expenditure. Although the number of participants, in particular of elderly subjects, may limit respective conclusions, we did not find indicators that the insulin effect was modified by sex or age. It was neither associated with unwanted side effects on sleep. Our results demonstrate that insulin delivered to the brain via the intranasal route exerts a long-lasting, behaviorally relevant effect on eating behavior. While the exact mediators behind such a delayed hypophagic action of enhanced brain insulin signaling cannot be derived from our data, this finding underlines the efficacy of intranasal insulin in curbing food intake in humans.

We have previously shown that intranasal insulin administered to young male subjects in the fasted state (Benedict et al., 2008) acutely decreases food intake, and reduces the intake of palatable snacks in young female subjects after lunch (Hallschmid et al., 2012). The latter effect concerned post-prandial snacking in the afternoon and likely resulted from effects on reward-processing pathways, which may be assumed to have also played a role in the present experiments, although our study design clearly does not allow for a differentiation between reward- and hunger-related aspects of eating. In the former experiment (Benedict et al., 2008), the hypophagic insulin effect in the young men was observed in the late morning and around 80 min after intranasal delivery of 160 IU insulin, i.e., the same dose as applied in the present experiments. It was found during ad-libitum intake from a breakfast buffet that was comparable in composition and size to the buffet offered in the present study. Insulin administration in the morning led to a macronutrient-unspecific reduction in calorie intake of around 190 kcal in young men, which exceeded the drop in food intake of 110 kcal found in the young male subjects of the present experiments. Both effects developed against the background of well-comparable overall calorie intake (1,350 vs. 1,330 kcal in the respective placebo conditions of the former and, for the young men, the present study). In the former study (Benedict et al., 2008), young female participants did not show an insulin-induced reduction in food intake whereas pre-sleep insulin delivery reduced breakfast size in the young women of the present experiments. There are some indicators that central nervous insulin, with regard to food intake, may be less efficient in female compared to male organisms (Clegg et al., 2003, 2006). However, the women of our previous study (Benedict et al., 2008) ate around 130 kcal less than their female counterparts of the present experiments (769 vs. 895 kcal in the respective placebo conditions), so that a biasing contribution of bottom effects cannot be excluded.

Basal energy expenditure assessed in the young subgroup before breakfast was comparable between conditions, indicating that the reduction in breakfast intake was not a compensatory effect, but yielded a net decrease in energy intake. This effect was primarily caused by a drop in the consumption of carbohydrates. This finding ties in with experiments in rats indicating that insulin administration to the CNS reduces sugar intake (Figlewicz et al., 2008) and suggests that, in accordance with the major role of peripheral insulin for blood glucose regulation, the anorexigenic effect of the hormone on the brain might focus on carbohydrates. However, the relative paucity of available data in humans (Benedict et al., 2008; Hallschmid et al., 2012; Jauch-Chara et al., 2012) and animals (Woods et al., 1979; McGowan et al., 1992; Air et al., 2002; Clegg et al., 2003, 2006) at the moment does not permit sound conclusions on macronutrient-specific effects of brain insulin on eating behavior (for review see Kullmann et al., 2016; Lee et al., 2016). Animal research has indicated that the adiposity signals insulin and leptin can directly act on the brain reward circuitry to decrease the intake of particularly palatable foods (Figlewicz et al., 2007). Although at a first glance, the insulin-induced reduction in carbohydrate intake would fit with the assumption that intranasal insulin specifically reduces the intake of highly rewarding foods (Hallschmid et al., 2012), sweet items as a food category were not differentially affected here. Considering that hunger ratings were comparable between conditions, it may be concluded that intranasal insulin did not affect hunger motivation, but rather acted via satiating factors that contribute to the termination of a meal. We did not find discernible treatment-induced changes in ghrelin and leptin, two hormones of paramount relevance for food intake control (Morton et al., 2014). A mild insulin-induced decrease in plasma glucose concentration apparently was restricted to the young participants although our study was not designed to detect respective differences between age groups. This dip in blood glucose, which was presumably due to absorption of insulin into the blood stream via the nasal mucosa (Ott et al., 2015), was statistically unrelated to the insulin-induced reduction in breakfast intake. It can be safely excluded to have affected breakfast intake because of its transient nature, and because nocturnal decreases in blood glucose levels rather increase than attenuate food intake in the morning (Schmid et al., 2008).

The inhibitory impact on ad-libitum food intake exerted by intranasal insulin emerged across 10 h, which is a surprisingly long period of time when compared to respective effects in related studies (Benedict et al., 2008; Hallschmid et al., 2012; Jauch-Chara et al., 2012). Considering that degradation of the insulin molecule in the central nervous compartment can be assumed to be relatively delayed in comparison to its half-life in the circulation of 4–6 min (Duckworth et al., 1998), the enhanced brain insulin signal may in principle be still functionally active after a prolonged period of time. This assumption is supported by the finding that shifts in direct current EEG potentials triggered by intranasal insulin do not yet reach their maximum after around 90 min of recording (Hallschmid et al., 2004b). Alternatively, an indirect mediation of the observed effect might be assumed, but cannot be derived from our data inasmuch as endocrine signals like ghrelin and leptin, but also indicators of sympathovagal balance were unchanged and our study does not warrant a strict differentiation between central and peripheral mediators. Intranasal insulin exerted its anorexigenic effect across an interval of nocturnal sleep which, in itself, has turned out to be a relevant modulator of food intake (Schmid et al., 2015). Total sleep deprivation for one night leads to activity changes in brain functions that favor food intake (Greer et al., 2013), and partial sleep deprivation of 4 h for one night strongly increases breakfast intake in healthy men (Brondel et al., 2010). Since we did not observe differences in total sleep time nor in sleep architecture between conditions, insulin presumably did not exert major effects on sleep. However, sleep might have prolonged the insulin effect by reducing interfering effects of external stimuli, or increases in hunger typically developing in awake subjects across extended periods of time.

The relative reduction in pre-breakfast hunger ratings in the elderly, as compared to young participants, might be related to decreasing responses to food cues (Cheah et al., 2013) and impaired dynamics of satiety-regulating factors (Rolls et al., 1995) that emerge during aging, but was not reflected in differences in actual food intake. Young and elderly participants were also equally responsive to the anorexigenic effect of insulin. However, probably because of the relatively small number of elderly participants the effect appeared to be less robust in this group so that further studies should substantiate this finding. While decreases in central nervous insulin sensitivity have been linked to cognitive deficits and Alzheimer's disease, pathologies associated with advanced age (Freiherr et al., 2013), our results suggest that, at least in healthy individuals, age does not independently affect the role of insulin in central nervous networks that control food intake. Likewise, insulin sensitivity in the body periphery is not independently affected by biological age as long there is no increase in fat mass (Karakelides et al., 2010). Thus, the good health status of our elderly participants, as verified by clinical examination and evidenced by their merely moderately elevated body weight in comparison to our young subjects, might have ensured sufficient potency of the insulin signal. Vice versa, indicators of high cerebral insulin sensitivity have been found to be associated with successful loss of body fat during lifestyle intervention (Tschritter et al., 2012).

## Conclusion

In sum, we demonstrate that intranasal insulin administration before nocturnal sleep elicits a reduction in breakfast intake in healthy subjects that is not compensated for by changes in energy expenditure. These findings suggest that insulin administered to the brain before sleep may potentiate the satiating effect of food intake in the next morning. The effect observed here was of moderate impact, and potential compensatory changes in eating behavior throughout the rest of the day were not investigated. In previous studies, four daily doses of intranasal insulin, one of them administered before going to bed, induced a reduction in body weight and fat in healthy participants (Hallschmid et al., 2004a), suggesting that on the long run the anorexigenic effect of (pre-sleep) intranasal insulin administration can affect body weight regulation. Thus, central nervous insulin administration regimens focusing on the sleep period may exert beneficial effects on metabolic health and might even help prevent or treat the brain insulin resistance associated with metabolic disorders (Kullmann et al., 2016).

## Author contributions

JS conducted the data analyses. MH collected the data and contributed to the analyses. Both authors interpreted the data and wrote the manuscript.

## Funding

This research was supported by grants from the Deutsche Forschungsgemeinschaft (DFG; SFB 654 “Plasticity and Sleep”), from the German Federal Ministry of Education and Research (BMBF) to the German Center for Diabetes Research (DZD e.V.; 01GI0925), and the Helmholtz Alliance ICEMED—Imaging and Curing Environmental Metabolic Diseases (ICEMED), through the Initiative and Networking Fund of the Helmholtz Association. The funding sources had no input in the design and conduct of this study; in the collection, analysis, and interpretation of the data; or in the preparation, review, or approval of the article.

### Conflict of interest statement

The authors declare that the research was conducted in the absence of any commercial or financial relationships that could be construed as a potential conflict of interest.

## Supplementary material

Supplementary Figure 1 **Boxplots of calorie intake from the breakfast buffet assessed in the morning after intranasal administration of placebo (vehicle; blue bars) and insulin (160 IU; red bars) at 2220 h of the preceding evening (A)** according to age groups (elderly subjects, darker shades; young subjects, brighter shades) and **(B)** according to sex.
