# Supplementary figures and images for: Central Nervous Insulin Administration before Nocturnal Sleep Decreases Breakfast Intake in Healthy Young and Elderly Subjects
Source: Front Neurosci. 2017 Feb 8;11:54. doi: 10.3389/fnins.2017.00054 (PMC5296307; doi:10.3389/fnins.2017.00054)

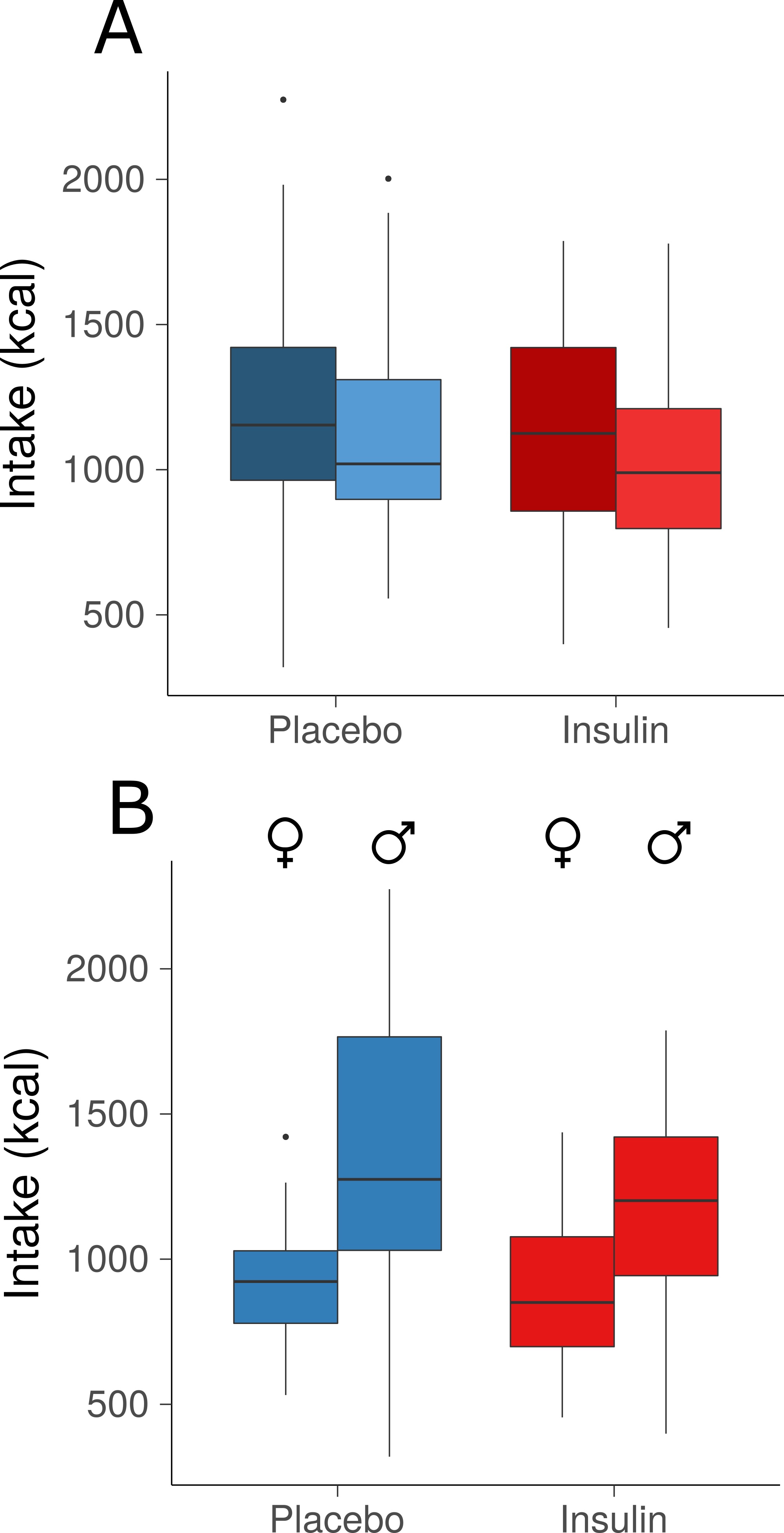

Supplement: Supplementary Figure 1 — Boxplots of calorie intake from the breakfast buffet assessed in the morning after intranasal administration of placebo (vehicle; blue bars) and insulin (160 IU; red bars) at 2220 h of the preceding evening (A) according to age groups (elderly subjects, darker shades; young subjects, brighter shades) and (B) according to sex. [file Image1.JPEG]
